# Connect Active Programme (CAP): A Pilot RCT to Enhance Physical Activity and Intergenerational Relationships Through Dyadic Digital Walking Exercises

**DOI:** 10.3390/healthcare13162043

**Published:** 2025-08-19

**Authors:** Mimi Mun Yee Tse, Percy Poo-see Tse, Ka Yan Ip, Ho Yuen Lam, Pak San Chong, Tyrone Tai On Kwok, Grace Yuying Sun, Samuel Kai Wah Chu, Kin Pong To

**Affiliations:** School of Nursing and Health Studies, Hong Kong Metropolitan University, Hong Kong; pptse@hkmu.edu.hk (P.P.-s.T.); s1335520@live.hkmu.edu.hk (K.Y.I.); s1167417@live.hkmu.edu.hk (H.Y.L.); s1263037@live.hkmu.edu.hk (P.S.C.); ttokwok@hkmu.edu.hk (T.T.O.K.); gsun@hkmu.edu.hk (G.Y.S.); skwchu@hkmu.edu.hk (S.K.W.C.); kpto@hkmu.edu.hk (K.P.T.)

**Keywords:** older adults, community-dwelling, healthy ageing, sedentary behaviour, intergenerational, technology acceptance

## Abstract

**Background/Objectives**: Sedentariness is a common phenomenon among both the elderly and the young in modern society. Changes in work structure, leisure activities, and technological advancements have contributed to excessive sitting time. To address sedentary lifestyles across generations, this randomised controlled pilot trial aimed to investigate the effectiveness of the Connective Active Programme (CAP) in improving intergenerational relationships, psychological well-being, and physical fitness among older adults. **Methods**: Twenty dyads of older and younger adults from the same family were recruited and randomly allocated to an experimental group or a control group in a 1:1 ratio. The experimental group participated in six weekly 2-h dyadic walking sessions supported by digital information and mobile applications, whereas the control group received weekly digital pamphlets. **Results**: Compared to the control group, the experimental group showed significant improvements in younger participants’ intergenerational relationship quality, as well as in older participants’ 6-Minute Walk Test performance and WHOQOL-BREF scores. **Conclusions**: The CAP appears to be a viable strategy to enhance intergenerational relationships and promote healthy ageing among community-dwelling older adults in Hong Kong.

## 1. Introduction

Older adults often experience a decline in their physical mobility as their ageing journey progresses. Indeed, their impaired physical mobility negatively affects their physical and psychosocial functions [1]. It reduces older adults’ motivation to move around or perform exercises, as well as their activity tolerance. Thus, physically inactive older adults are at an elevated risk of experiencing all-cause and cardiovascular mortality, prostate cancer and breast cancer, falls, ADL disability, Alzheimer’s disease, and depression, which lowers their quality of life [2]. Also, functional limitations prevent older adults from travelling to far places, which restricts their social circles and increases their loneliness.

Not only do older adults lack physical activity (PA), but younger generations also struggle with a similar situation [3]. These young individuals often find themselves entangled in hectic schedules, multiple commitments, and various responsibilities in this fast-paced and demanding society. Their pressures at work and school and other personal obligations consume most of their time and attention, leaving little or no time for physical exercise [4]. Following a World Health Organisation-led study, a significant proportion, greater than 80%, of school-attending adolescents worldwide did not satisfy the current recommendations of at least an hour of PA each day [5]. Thus, it is crucial to acknowledge the importance of exercise for their physical and mental health. Regular exercise is beneficial to all generations, as it helps produce and maintain healthy bones, muscles, and joints; lowers the risk of developing diabetes, dying from heart disease, and developing colon cancer; reduces feelings of anxiety and depression; and fosters psychological well-being [6]. It is advantageous when younger and older generations perform physical, intergenerational activities together [7].

Considering the everyday sedentary lifestyles of both generations, they could exercise together to motivate each other. Extensive research has shown that exercising with a partner could foster better physical and mental health [8,9]. A systematic literature review by Napetschnig et al. (2025) identified essential technical, social, and psychological requirements for successful technology use in promoting physical activity among older adults. These include user-friendly interfaces, motivational elements like gamification, social interaction opportunities, accommodation of mobility limitations, and data privacy measures. The study emphasises a holistic approach to technology development to improve acceptance, effectiveness, and health outcomes in seniors [10].

Dyadic exercise arrangement also opens the door for intergenerational learning and wisdom/knowledge exchange [11]. This reciprocal learning positively affects younger generations concerning their sense of purpose in life and attitudes toward and comfort when communicating with older adults [12]. It also breaks down age-related barriers and promotes a sense of harmony. Through this intergenerational approach, older adults could benefit from the younger generation’s energy and enthusiasm. This encouragement may lead them to participate in more physical activities and maintain their health status.

There is a growing body of literature that demonstrates great acceptance of smartphones among older adults [13,14]. While older adults may not be as technologically savvy in general, younger adults can certainly come to the rescue in helping them adapt to this technology-driven society. These young people can use their expertise in digital technology to enhance digital literacy among older adults, as many of these younger adults spend more than 2 h of recreational screen time daily [3]. Younger adults could act as digital buddies and enhance digital literacy among older adults [15]. A 2023 meta-analysis showed that digital health literacy (DHL) interventions among older adults, delivered via face-to-face or web-based education, effectively improve eHealth literacy, self-efficacy, and digital skills. Face-to-face methods were particularly effective, partly because they support social interaction, an important aspect that can be fostered by intergenerational support. These interventions typically cover computer basics, health portal use, and peer support forums [16]. With the support from younger generations, perhaps older generations would feel more confident using mobile technology independently. Both generations could also explore new mobile apps and their functions together to bond and create conversations.

Despite the long-standing history of intergenerational programmes, most empirical research showcasing the effects of intergenerational interactions on older adults’ health-related outcomes has only started to emerge within the past two decades, with most studies conducted after the year 2000 [17,18]. Existing evidence has revealed notable research gaps that require further investigation [19]. First, most studies emphasise non-familial intergenerational relationships [20,21]. Second, researchers prioritised studies involving younger children and their grandparents, mainly in a caregiver–care recipient relationship due to the absence of parents at home [22,23,24]. In contrast, research that includes younger adults aged 18 or above is limited. Locally, the majority of intergenerational studies have focused on cultural and knowledge exchange [11], and research on technology-based intergenerational exercise programmes is even more scarce, except for one similar study that emphasised mental well-being [15]. Additionally, recent studies have underlined the role of co-learning and co-navigation of mobile technologies in fostering intergenerational engagement and health behaviour change [25].

Given that older adults are inactive and less likely to explore new places, and younger adults tend to be more technologically savvy, this present study introduced the Connect Active Programme (CAP), a walking exercise programme using mobile apps to bridge the generational gap and promote intergenerational activities. The CAP paired an older adult with their younger family member/relative as a “dyad”. By practising walking exercises together, older and younger generations could build healthy intergenerational relationships and maintain better physical and psychological well-being. Simultaneously, older adults could gain practical digital skills when younger adults navigate the mobile apps suggested in the programme.

The Connect Active Programme (CAP) aligns with Hong Kong’s Smart Ageing Blueprint by promoting digital literacy, physical activity, and intergenerational engagement—key priorities in advancing active ageing through innovation [26]. It also reflects the WHO’s Decade of Healthy Ageing framework by enhancing both intrinsic capacity and supportive environments, in line with global recommendations to maintain functional ability and reduce social isolation among older adults [27]. The CAP’s design supports local and global strategies for healthy ageing through cost-effective, community-based interventions.

Addressing the shared challenge of sedentary behaviour in older and younger generations, the CAP innovatively combines familial intergenerational engagement with technology-enhanced walking exercises. This approach not only promotes physical activity but also fosters meaningful family interactions, setting it apart from programmes that may focus solely on one generation or use technology in a less integrated manner.

### 1.1. Conceptual Framework

**Figure 1:**
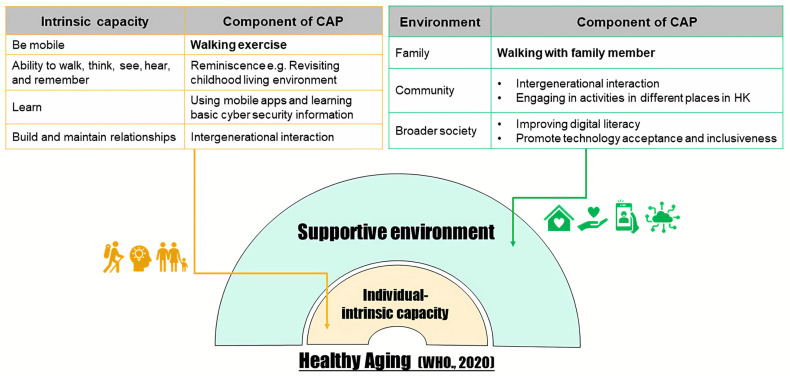
Conceptual Framework of the Connect Active Program (CAP).

The developed CAP was underpinned by the conceptual framework of healthy ageing from the World Health Organisation, which calls for actions from various sectors to enable older adults to remain an asset to the family, communities, and economies [28]. The intervention was designed to maintain and improve older adults’ functional ability, which is the core purpose of healthy ageing, through two dimensions—the intrinsic capacity and the environment (see Figure 1 for details).

### 1.2. Research Aims

The overall objectives of this study were to examine the effectiveness of the Connect Active Programme (CAP) in improving intergenerational relationships, psychological well-being, and physical fitness for older adults. Therefore, this pilot study aimed to investigate the feasibility and preliminary effectiveness of the Connect Active Programme (CAP) in improving intergenerational relationships, psychological well-being, and physical fitness among older adults and their younger family members.

## 2. Methods

### 2.1. Study Design & Procedures

**Figure 2:**
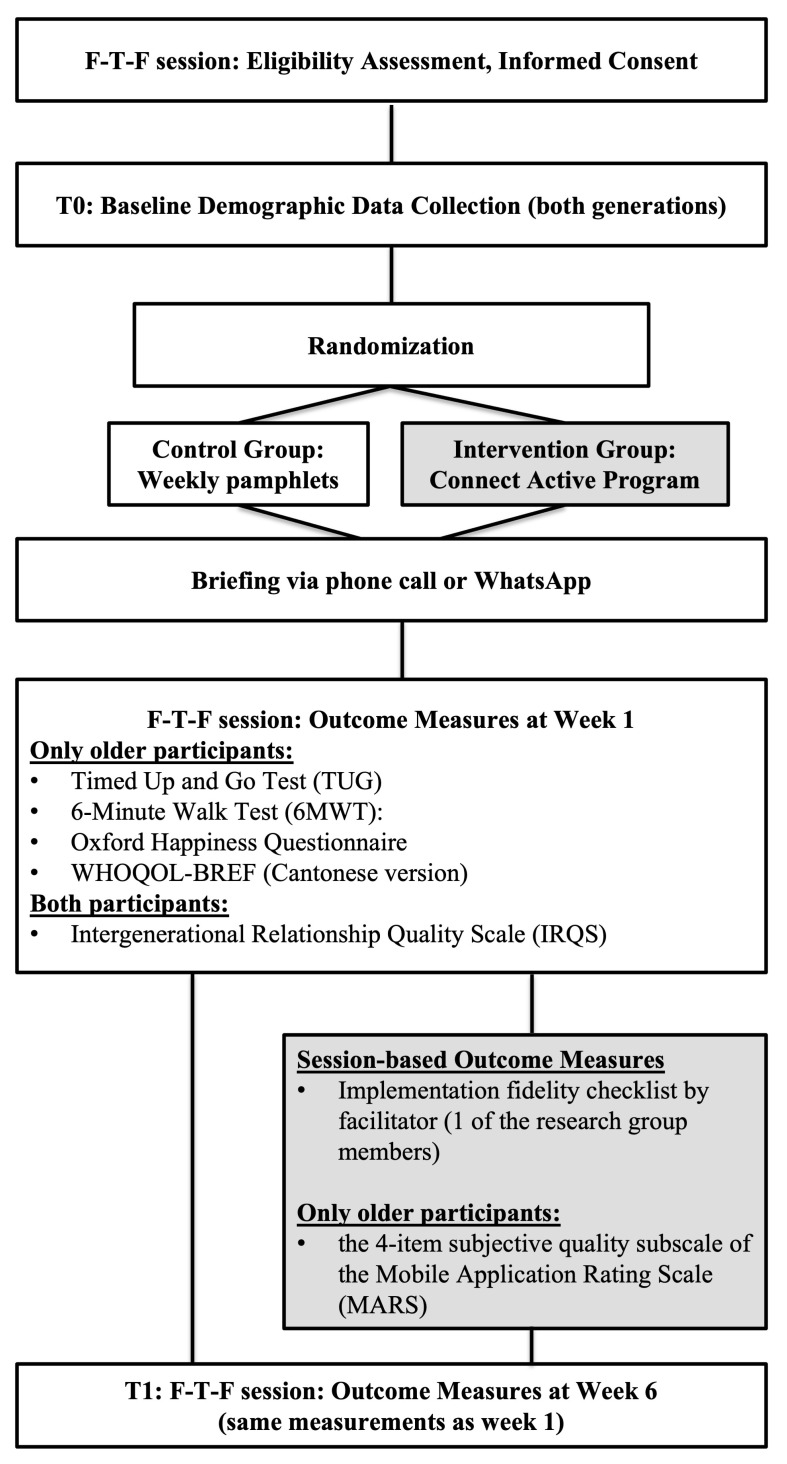
Schema of the Study Design.

This study employed a two-armed pilot randomised controlled trial. All methods were performed following the relevant guidelines and regulations of the Hong Kong Metropolitan University Research Ethics Committee (REC Reference Number: HE-RD/2023/2.19). The date of the first trial registration was 11 January 2024 (ClinicalTrials.gov registration number: NCT06202482). Figure 2 illustrates the flow of this study (see Figure 2 for details). Before the commencement of the CAP, the research team conducted face-to-face eligibility assessments on the prospective dyads who were fully informed and had already signed the written consent form. The team then collected baseline demographic information on the dyads. Finally, the dyads were randomised into either the intervention group or the control group.

Eligibility screening was conducted by trained research assistants who followed a standardised protocol to ensure consistency across assessments. To maintain inter-rater reliability, the research assistants underwent rigorous training and participated in regular calibration meetings, ensuring that all assessors applied the eligibility criteria uniformly. Additionally, informed consent was obtained from all participants during face-to-face sessions before their inclusion in the study, adhering to ethical research standards. Attrition was limited to one dyad withdrawing due to scheduling conflicts, and their data were excluded from analysis. Missing data were managed using pairwise deletion to preserve dataset integrity.

### 2.2. Participants and Sampling

The research team recruited 20 dyads (40 participants) through convenience sampling. The location of data collection was according to each week’s theme (see Supplementary Table S1). This sample size was based on the recruitment feasibility and previous publications [29,30]. As a pilot study, the sample size of 20 dyads was based on feasibility and was consistent with the last exploratory trials in similar settings [24,25]. While formal power calculations were not conducted, this sample size was deemed adequate for preliminary assessment. An information poster was distributed electronically, and written informed consent was obtained before the intervention.

Each dyad comprised an older participant, age 55 or above, and a younger adult between the ages of 18 and 45 from the same family (e.g., parents, grandparents, or relatives). All participants had to be able to understand Cantonese and written Chinese, own a smartphone with access to a network connection, and partake in walking and stretching exercises. Those who had been diagnosed with a mental disorder by a neurologist or psychiatrist and had a history of drug addiction were excluded from this study.

Given the pilot nature of this trial, we prioritised feasibility and participant engagement. The sample size was determined based on recruitment feasibility and previous similar studies, aiming to provide initial insights rather than definitive conclusions.

This study utilised convenience sampling to recruit participants from a specific community, which facilitated practical data collection. However, this approach limited the generalisability of the findings due to potential selection bias and reduced external validity. We acknowledge this limitation and suggest that future research incorporate more robust sampling techniques, such as stratified or random sampling, to improve the representativeness of the sample and strengthen the applicability of the results.

This study employed convenience sampling, which may limit the generalisability of the findings to the broader population. As participants were recruited from a specific community and may share similar characteristics, the results may not fully reflect the experiences of more diverse or randomly selected populations. Future studies should consider employing probabilistic sampling methods to enhance external validity.

As a pilot study, formal power calculations were not conducted a priori. The sample size of 20 dyads was based on feasibility and aligned with comparable exploratory trials. A post hoc power analysis and specification of minimal clinically important differences (MCIDs) for primary outcomes—e.g., approximately 30 m for the 6MWT and 10% change in WHOQOL-BREF domain scores—will be considered in future definitive trials to ensure adequate statistical power.

### 2.3. Randomisation, Allocation Concealment, and Blinding

This study employed simple randomisation to allocate all 20 dyads (40 participants). Each participant was assigned to a group by opening a sequentially numbered, opaque, sealed envelope that contained a card indicating their group assignment. A person independent of the study team generated the group allocation to achieve allocation concealment. Blinding was likely impossible because the intervention had been disclosed in the recruitment poster.

Due to the nature of the intervention, the blinding of outcome assessors was not feasible. This limitation may have introduced detection bias, particularly in self-reported outcomes, and should be considered when interpreting the results.

Although allocation concealment was ensured through sealed envelopes and third-party randomisation, the blinding of outcome assessors was not feasible due to the nature of the intervention. To reduce potential bias, objective outcome measures such as the 6-Minute Walk Test were included.

Post-intervention assessors were not blinded due to logistical and practical constraints. To mitigate potential expectation bias, standardised assessment protocols were used for all outcome measures, and objective measures (e.g., 6MWT) were prioritised where feasible.

### 2.4. Intervention

Dyads in the intervention group participated in a 6-week Connect Active Programme (CAP)—the details of which can be found in Supplementary Table S1. Weekly walking themes (e.g., sports ground walk, park walk) were co-designed with participants. Apps included pedometer trackers, route planners, and health diaries selected for simplicity and relevance to older users. The research team designated a theme for each week’s walking exercise, either indoors or outdoors. This study also took a co-design approach, involving both the participants and the research team when choosing the location of the venues. This discussion was conducted via phone call or WhatsApp. For participants’ preparation, weekly supplementary information (pamphlets) about the upcoming activities was distributed to the dyads at the end of each session.

The mobile apps used in the intervention included pedometer trackers (e.g., Nike Run Club), route planning apps (e.g., Google Maps, Hong Kong Hiking Routes), health journals (e.g., Meitu for photo logs), and communication tools (e.g., WhatsApp). These apps were standardised across participants in the intervention group. Prior to the first session, participants received a brief orientation on app usage through a face-to-face demonstration and written instructions. While no specific digital tasks were mandated, participants were encouraged to log their walking routes and steps weekly using the apps. To ensure intervention fidelity, facilitators conducted weekly check-ins and monitored adherence via self-reported walking logs and app screenshots submitted by participants. Engagement was assessed through session attendance records and qualitative feedback on app use, with the app ratings summarised using the Mobile Application Rating Scale (MARS).

The CAP intervention involved weekly 2 h sessions over six weeks, each with themed walking routes (e.g., park, sports ground) and light stretching routines. Weekly goals (e.g., step counts, distance targets) were co-designed with participants, and digital literacy was informally developed through guided use of mobile apps. A full description of the CAP’s weekly structure and walking routines is provided in Supplementary Table S1.

### 2.5. Control Group

Participants in the control group received weekly leaflets that were distributed through WhatsApp as usual care. The pamphlets contained information about healthy diets recommended by the local authority. The research team arranged in-person assessment sessions at two time points—during the first week (baseline) and in the sixth week (post-intervention).

The control group received weekly digital pamphlets on healthy diets as usual care, without structured physical activity or social engagement. While this design is acceptable for a pilot study, we acknowledge that the absence of an active placebo or attention-matched condition may have introduced placebo or attention bias, particularly for psychological outcomes. Future trials should consider incorporating an active control involving light social interaction or non-exercise activities to isolate the specific effects of the intervention better.

We recognise that the control group received only educational pamphlets without interactive components, which may have resulted in differential engagement and introduced bias. This limitation should be addressed in future studies by designing a more active control condition that matches the intervention group in attention and social interaction.

We acknowledge that the control group received digital educational materials only, while the intervention group participated in weekly in-person sessions involving physical and social interaction. This unequal attention may have influenced outcomes such as psychological well-being through non-specific effects. Future trials should include an active control condition with comparable contact time to isolate intervention effects better.

### 2.6. Descriptive Measures

Participants received an electronic questionnaire to input demographic data. Socioeconomic and demographic information was collected as age, gender, the highest level of education, the nature of one’s job, monthly income, lifestyles (exercise, drinking, and smoking habits), health history (chronic illness), and level of dependency. Additionally, questions related to the exclusion criteria (e.g., whether having a phone with a network connection or drug abuse) were included to confirm the eligibility of the participants.

While basic socioeconomic and lifestyle data were collected through a self-developed electronic questionnaire, future studies will consider validated tools such as the International Physical Activity Questionnaire (IPAQ) and the Physical Activity Scale for the Elderly (PASE) to enhance the reliability of physical activity assessments. Anonymity and confidentiality were maintained throughout the study: data were stored in password-protected files accessible only to the research team, and participants’ identities were de-identified before analysis, as approved by the ethics committee.

### 2.7. Outcome Measures

Participants completed the assessments and questionnaires before (baseline-T0) and upon completion (week 6-T1) of the CAP.

#### 2.7.1. Primary Outcome

The 6-Minute Walk Test (6MWT) (measured in minutes) was initially created to assist in the examination of cardiopulmonary patients and was developed by the American Thoracic Society. It is a light-intensity exercise test to evaluate aerobic capacity, endurance, and oxygen saturation [31]. The 6-Minute Walk Test (6MWT) was conducted in a flat, indoor environment under standardised conditions, following the American Thoracic Society’s guidelines.

#### 2.7.2. Secondary Outcomes

1. Time Up and Go (TUG) (measured in seconds) was used to assess mobility, movement, static balance, and dynamic balance in people with musculoskeletal impairments and conditions related to ageing, as well as quality of life and pain [32]. The instructions were given as follows: standing up from a chair, walking 3 m, turning, and sitting back down.
2. Quality of life was evaluated utilising the World Health Organisation Quality of Life-BREF (WHOQOL-BREF). It contains four realms: physical health, psychological health, social relationships, and environment. A 5-point response scale was used in the scale [33]. WHOQOL-BREF has good validity and reliability [34].
3. The Oxford Happiness Questionnaire is a 29-item measure of happiness, from 1, “strongly agree”, to 6, “strongly disagree”. The scale’s reliability was 0.91 [35]. Scores on the Oxford Happiness Questionnaire were not reversed; lower scores indicate higher levels of happiness, as per the original scoring guidelines.
4. The intergenerational relationship quality scale (IRQS) was used to appraise the changes in intergenerational relationships. This questionnaire allowed the older participants to self-rate the relationship between the two generations. A five-point response scale was applied in the scale [36]. The Intergenerational Relationship Quality Scale (IRQS) has demonstrated good reliability and validity in prior studies involving older Chinese adults (Cronbach’s alpha = 0.86), making it suitable for our study population.
5. The four-item subjective quality subscale of the Mobile Application Rating Scale (MARS) was deployed to assess the overall quality of mobile apps involved in this study [37]. MARS incorporates four objective quality subscales (engagement, functionality, aesthetics, and information quality) and one subjective quality subscale. Since a lengthy questionnaire would be complicated for the older participants to complete, the research team only adopted the subjective quality subscale. All four items were assessed on a five-point scale. The subjective quality subscale showed excellent internal consistency (alpha = 0.93) and inter-rater reliability, with an intraclass correlation coefficient (ICC = 0.83). Higher scores indicate a higher level of subjective quality. The subjective quality subscale of the Mobile Application Rating Scale (MARS) was self-administered by older participants in the intervention group to capture user perspectives on app quality.

### 2.8. Data Analysis

To conduct statistical analyses, the IBM-SPSS version 28 was utilised. The research team applied descriptive statistics (frequency %; mean (standard deviation)) to explain the demographic data. Concerning the smaller sample size, the non-parametric Mann–Whitney U-test was performed to compare outcomes between the intervention and control groups [38,39]. A *p*-value of <0.05 was considered statistically significant. In addition to *p*-values, we recommend reporting effect sizes (e.g., r or Cohen’s d) and confidence intervals to provide readers with a better understanding of the magnitude and practical significance of observed differences.

As a pilot study, a sample of 20 dyads (40 participants) was selected based on feasibility and consistency with similar exploratory trials. Although no formal power analysis was conducted, this size allowed for the assessment of recruitment, retention, and initial effect trends. These findings will inform the design and power calculations of future large-scale, definitive trials.

Effect sizes (e.g., Mann–Whitney r) and 95% confidence intervals have been added alongside *p*-values to enhance interpretability. All significance levels have been reported using exact *p*-values (e.g., *p* = 0.015) rather than threshold-based notation, and formatting has been standardised across text and tables.

## 3. Results

The demographic characteristics of older and younger participants are displayed in Table 1 and Table 2, respectively. There were 20 older adults and 20 younger adults in the study, and they were randomly assigned to the experimental and control groups, with 10 dyads in each group. No dropouts occurred during the 6-week intervention. Further, all participants attended all walking exercise sessions during the 14-week intervention stage (from January 2024 to March 2024), thereby demonstrating a 100% participation rate. The trial concluded at the end of the intervention stage. Table 1 shows that the mean age of the older participants was 67.6 in the experimental group and 66.7 in the control group. Though the older participants in both groups were educated, the levels varied in both the experimental and control groups. The proportion is similar in terms of having or not having any exercise habits. Considering smoking habits, only one participant in the experimental group sometimes smokes. Thirty per cent of participants reported a good past health history, whereas half had two or more chronic illnesses. Despite this, all older participants were independent in their daily lives.

Table 2 shows the demographic characteristics of the younger participants. The mean age was 28.6 in the experimental group and 30.0 in the control group. In both groups, 60% were females with an educational level of secondary school or above. In terms of employment, 90% worked full-time and 10% part-time. However, the monthly income was inconsistent: 30% earned ≤HKD 10,000 or less, while the majority (70%) had a monthly income of HKD 20,001 or above. From a lifestyle perspective, the experimental group showed that 70% had exercise habits and 40% had occasional drinking habits. On the other hand, the control group comprised 50% of respondents with exercise habits and 20% of occasional drinkers. Comparing the two groups, 100% of the younger participants were non-smokers, without chronic illness, and independently living.

As seen in Table 3, the analyses revealed statistically significant differences in the scores obtained from various outcome measures. First, the 6-Minute Walk Test (6MWT) results exhibited a statistically significant between-group difference (*p* = 0.005). Further, the scores derived from the WHOQOL-BREF (Cantonese version) showed a statistically significant change (*p* = 0.015). Moreover, the Intergenerational Relationship Quality Scale (IRQS) scores for the younger participants demonstrated statistically significant differences between the two groups (*p* < 0.001). These findings indicated that the intervention significantly improved intergenerational relationships perceived by the younger participants and the physical and psychological well-being of the older participants, as evidenced by the *p*-values falling below the predetermined significance level of 0.05.

Meanwhile, no statistical differences were observed in the measurement of the Time Up and Go Test (*p* = 0.123), the Oxford Happiness Questionnaire (*p* = 0.393), and the Intergenerational Quality Scale of the older participants (*p* = 0.315). This discrepancy in intergenerational perception warrants discussion. Possible factors include generational expectations, communication styles, or differences in how relational changes are perceived.

This study collected quantitative participant feedback utilising the Mobile Application Rating Scale, as shown in Table 4. This scale facilitated a session-based evaluation completed by the older generation in the experimental group, which assessed their mobile app usage over 12 months. The scale also captured their willingness to pay for the mobile app and their intention to recommend it to others. The ratings for the four items on the scale, scored on a scale from 1 to 5, were averaged and subsequently calculated. The analysis of these ratings revealed that five of the seven mobile apps employed in the experimental groups received average scores below 2.5, suggesting a relatively low perceived quality of these apps among the older participants. This result indicates that despite the availability of younger peer support, the acceptance of mobile apps and their perceived quality can fluctuate among older adults. Qualitative feedback or brief participant comments could help explain why certain apps were less favourably rated—e.g., usability issues, interface design, language barriers, or feature irrelevance.

All participants in the intervention group attended the six scheduled CAP sessions, resulting in 100% adherence. While detailed step count or MET-minute data were not systematically collected, future studies will incorporate digital tracking metrics to quantify physical activity dose and explore dose-response relationships.

## 4. Discussion

This present study enrolled 40 participants, both older and younger adults, forming 10 dyads each in the experimental and control groups, respectively. The main findings indicated that the scores for the 6-Minute Walk Test, the WHOQOL-BREF, and the Intergenerational Relationships Quality Scale of the younger participants significantly improved in the experimental group compared to the control group upon completion of the CAP. This intergenerational approach could enhance the physical and psychological well-being of older community-dwelling adults in Hong Kong.

The results of the 6-Minute Walk Test revealed significant improvement in between-group comparisons. This positive outcome aligned with a systematic literature review of intergenerational interventions and their impacts on older adults’ health outcomes [40]. Through the CAP, the pervasive issue of sedentariness in both older and younger adults was addressed. Indeed, it has become widely evident that a sedentary lifestyle contributes to the promotion of premature ageing and increased mortality risks [41,42]. Yet, PA levels remain low in these two age groups despite the alarming numbers of sedentariness-induced health issues. Regular PA is one way to break sedentary patterns, but a common experience is the lack of motivation that prevents people from participating further [43]. A previous study suggested interventions involving reflective motivational processes, such as formulating detailed plans (specific time, location, and type of PA), to reduce sitting behaviour could be effective [44].

Additionally, existing research recognises the critical role of appropriate assessment tool selection [45]. Assessing the PA participation of older participants could allow researchers to tailor interventions that suit their needs and schedules by analysing their PA habits and sedentariness degree [46,47]. Measuring participants’ intrinsic motivation and perceived self-efficacy of their physical abilities could enable researchers to understand further how to increase participants’ willingness to be active [48]. These are crucial determinants of behaviour change, and further research is needed to investigate the effectiveness of the CAP using diverse measurement tools in improving specific health outcomes to achieve healthy ageing.

Recent evidence has shed light on the relationship between sedentary behaviour and brain health, warning the public about how prolonged sitting with inadequate physical exercise could destroy people’s brains [49]. Sedentary behaviour can have profound and wide-ranging effects on brain volume, connectivity, and cognitive functions, leading to an increased risk of dementia [50,51]. Older adults tend to spend their leisure time in sedentary positions [52], and many would choose walking for pleasure to compensate for sedentary time, given their limited physical agility or access to exercise facilities [53]. Another study further argues that PA, such as walking, should be the primary intervention for individuals suffering from chronic pain with a long life expectancy [54]. However, walking intensity should be a significant consideration for older adults who only walk as their regular PA, as a previous study reported that a slow walking speed was linked to a higher risk of dementia development [55]. While power walking has become a popular rehabilitation and exercise technique [56,57], its adoption among older adults as leisure PA remains limited, and much uncertainty still exists about its effectiveness in improving physical outcomes in this age group.

Far-reaching benefits from regular PA can be achieved when both generations start as soon as possible. Starting from the top of the body, the brain benefits greatly from consistent PA, simply by walking. One interesting finding from a recent study was that exercise therapy could alter brain functional connectivity, improving clinical symptoms and motor abilities in fibromyalgia patients who live with chronic, widespread pain [58]. Another important finding was that aerobic exercise (walking and dancing) could help regenerate a degree of plasticity in the white matter regions that are susceptible to age-related declines [59]. Plus, replacing sedentary leisure-time behaviour with PA could lower the risk of dementia [60]. The location to perform PA is a critical component to consider, as suggested by the latest research, which suggests that walking in nature could confer greater physiological benefits compared to artificial environments [61]. The physiological benefits of walking include the risk reduction of chronic age-related diseases such as cardiovascular and cerebrovascular diseases, hypertension, type 2 diabetes mellitus, and cancer, as well as the improvement of pain and function in musculoskeletal disorders, the promotion of sleep and mental health, and resilience boosts [53]. The CAP content, which involved walking outdoors and hiking, echoed the evidence from these latest findings.

Reviewing the current research on intergenerational intervention, the dominant younger party mainly focused on children. To address this gap, our preliminary data yielded insights regarding the significant positive changes in intergenerational relationships perceived by the younger generation aged 18 to 45. This is also in accord with previous research, which has demonstrated that younger generations also exhibit acceptance and interest in intergenerational programmes [62,63]. From the perspective of these younger participants, intergenerational activities could allow them to develop meaningful relationships with older adults and grow their sense of community engagement [62,63]. The intergenerational nature of the CAP encouraged the younger participants to initiate intergenerational interactions with their related older participants by guiding them to use mobile apps. These interactions could empower older adults to catch up to the rapid digital transformation and further lay the foundation for digital health, which matches the World Health Organisation’s goal to promote the adoption and scale-up of digital health and innovation [64].

In contrast to the present finding of the nonsignificant results of intergenerational relationships rated by the older participants, a previous study demonstrated that older adults exhibit a willingness to participate in intergenerational physical activity [47]. At the same time, they tend to be less intrinsically motivated for and less likely to enjoy general physical activities due to physical decline [47]. The timeframe of this present study may not have been sufficient for older adults to develop meaningful intergenerational bonds with younger adults. Also, the digital knowledge transfer from younger to older participants appeared to be more one-way learning than reciprocal learning, as mentioned in the literature review. Older adults tend to have an intrinsic and generative desire to offer guidance to younger generations to achieve greater things in life [65,66]. Thus, future research could emphasise the reciprocal learning element of the intergenerational programme to fulfil the human need for social exchange, which is essential in developing relationships [67]. Adding this fundamental component of reciprocal learning can promote a greater sense of self and purpose [66], which in turn can lead to better psychological outcomes in both younger and older generations [68].

While it is difficult to start and maintain a behaviour change, the social opportunities offered in the intergenerational intervention could motivate participants to continue with the newly developed behaviour [61]. As recommended in a new study proposal introducing the intergenerational Taekwondo programme, adding a cultural twist to the intergenerational intervention could raise both older and younger participants’ interest in participating, thus prolonging the new habit [69]. Intergenerational activities could create a sense of meaningfulness and mutual links between the two generations, which could then lower a feeling of loneliness [70]. When both generations have a common goal, such as sustaining physical activity performance, it could motivate them to continue this behaviour and allow them to feel supported [71].

Overall, the average scores of most mobile apps are below the equilibrium. Older adults tend to rate higher for more commonly used apps such as WhatsApp. Such hypothetical evidence corroborates the need to continue identifying the correlation between familiarity and perceived quality, as well as the factors underlying the acceptance and adoption of digital technologies by older adults. Moreover, according to the questionnaire, most seniors will not pay for mobile apps. It was an especially noteworthy finding that raised concerns about the deployment of financial resources for sustaining mobile app innovation targeting senior users.

The CAP provided a vital opportunity to advance the understanding of using an intergenerational approach to promote physical activity and reduce sedentary behaviour in both younger and older generations. The physical activities in the CAP are generally cost-effective and straightforward, and therefore, translating our findings into everyday practice is feasible. We should continue to explore novel, inexpensive intergenerational physical activities to bring out better health outcomes in both age groups. As a pilot study, we encountered challenges related to the environment, participants, and time, which may inform future replication with larger-scale research. Environmental constraints can significantly impact the field of exercise and physical activity. Our team faced constraints, including adverse weather conditions, such as freezing temperatures or heavy rainfall. Notably, these environmental factors are distinctively challenging to the senior participants’ ability to engage in physical activity, as they may limit outdoor exercise opportunities or discourage the family from venturing outside altogether. Consequently, researchers and practitioners need to consider alternative exercise options or indoor facilities to accommodate participants and ensure the continuity of their participation in the intervention. Furthermore, the intergenerational approach of the CAP has a potentially long-term impact, allowing young adults to take a path towards a healthy lifestyle early, which better prepares them for their ageing journey in the future. Thus, our findings should make an essential contribution to the field of ageing.

Future studies should expand measurement tools and explore other health outcomes. Researchers could conduct upscaled studies on older adults’ app preferences and develop apps with a co-design approach, which can increase their interest and persistence in physical activity participation. Taking individual factors such as chronic pain or a lower incentive to exercise into account when designing the intervention is also crucial. Recalling one of the intervention sessions in week 5, some of the older adults reported knee pain and fatigue. Our group’s efforts focused on assessing the client’s condition, providing health education on walking exercises, and arranging beginner-level hiking trails that ensured safety and allowed for continued activity. Likewise, both researchers and participants can derive stress from time constraints. Several older adults expressed overwhelming assessments at weeks 1 and 6. To mitigate this, it is recommended to carefully plan and communicate the assessment schedules, allow for flexibility, and provide clear instructions and information related to the assessments to minimise participants’ stress levels before the research implementation.

The strengths of our study include the provision of preliminary data and valuable insights. As an initial exploration of the intervention’s feasibility and potential effectiveness, our study is expected to identify potential issues or challenges and make necessary adjustments before future scale-up replication. Concurrently, randomised controlled trials, despite a relatively small sample size (*N* = 20 dyads), should be considered as a more rigorous evaluation of the intervention’s effectiveness upon the minimisation of confounding variables. However, our study still presents some limitations. The statistical power may be limited due to a smaller sample. Consequently, it may fail to detect significant effects, and caution should be exercised when interpreting the results. Further studies with larger sample sizes are suggested to confirm and generalise the findings. Another limitation stems from the questionnaires’ reliance on self-report measures, which might lead to susceptibility to biases, such as memory limitations or social desirability. Third, accompanying the dyads throughout the intervention, the facilitator occasionally received qualitative feedback. Selecting a simplified quantitative measure for technology acceptance can simultaneously limit comprehensive insights, such as the underlying reasons for lower user satisfaction with mobile apps. Conceptually, abundant studies highlighted the importance of investigating not only the quantity but also the quality of intergenerational contact. For example, the reduction of ageist attitudes among college students was found to be related to the quality, but not the frequency, of interaction [72]. In the same vein, a survey of university students added that good-quality contact was associated with more positive behavioural intentions toward older adults [73]. Henceforth, complementing this study with a qualitative research approach in future replication should be viable and significant.

As a pilot study, our findings should be interpreted with caution due to the small sample size and potential biases. However, the significant improvements observed in specific outcomes suggest that the CAP has promise and warrants further investigation in a larger, more rigorous trial. Future studies should also explore the long-term effects of such interventions and consider incorporating qualitative methods to gain deeper insight into the participants’ experiences.

This study included only immediate pre- and post-intervention assessments, with no follow-up to assess the sustainability of the effects. Future research should incorporate short- and medium-term follow-ups to evaluate the retention of physical and psychological benefits.

## 5. Conclusions

This pilot study found significant potential of the CAP in enhancing intergenerational relationships perceived by the younger generation and promoting physical and psychological well-being among older adults. Our findings suggest feasible and practical strategies that the general population can adopt to increase physical activity. These alternatives aim to prevent inactivity and ageing-related diseases and reduce mortality risks.

While the findings from this pilot study are promising, they should be interpreted with caution due to methodological limitations. Future large-scale, rigorously powered randomised controlled trials with active control conditions and extended follow-up periods are warranted to validate and expand upon these results.

## Figures and Tables

**Table 1:** Demographic characteristics of older participants (*N* = 20).

Demographics		Experimental (*n* = 10)		Control (*n* = 10)	
Age		M	SD	M	SD
		67.6	6.8	66.7	7.2
		*n*	%	*n*	%
Gender	Male	5	50	5	50
	Female	5	50	5	50
Education level	Below primary	2	20	2	20
	Primary	2	20	2	20
	Secondary	4	40	5	50
	Tertiary or above	2	20	1	10
Nature of the job	Full-time	2	20	2	20
	Part-time	0	0	0	0
	Unemployed	8	80	8	80
Monthly income (HKD)	≤10,000	6	60	7	70
	10,001–20,000	3	30	3	30
	20,001–30,000	1	10	0	0
	>30,000	0	0	0	0
Exercise habit	Yes	5	50	4	40
	No	5	50	6	60
Drinking habit	Never	6	60	10	100
	Sometimes	4	40	0	0
	≥3 days per week	0	0	0	0
	Daily	0	0	0	0
Smoking habit	Never	9	90	10	100
	Sometimes	1	10	0	0
	≥3 days per week	0	0	0	0
	Daily	0	0	0	0
Chronic illness	0	3	30	3	30
	1	2	20	2	20
	≥2	5	50	5	50
Dependency	Independent	10	100	10	100
	Dependent	0	0	0	0

M = mean, SD = standard deviation.

**Table 2:** Demographic characteristics of younger participants (*N* = 20).

Demographics		Experimental (*n* = 10)		Control (*n* = 10)	
Age		M	SD	M	SD
		28.6	7.2	30.0	6.5
		*n*	*%*	*n*	*%*
Dyadic relationship	Parent–child	10	100	10	100
Gender	Male	4	40	4	40
	Female	6	60	6	60
Education level	Below primary	0	0	0	0
	Primary	0	0	0	0
	Secondary	1	10	1	10
	Tertiary or above	9	90	9	90
Nature of the job	Full-time	9	90	9	90
	Part-time	1	10	1	10
	Unemployed	0	0	0	0
Monthly income (HKD)	≤10,000	3	30	3	30
	10,001–20,000	0	0	0	0
	20,001–30,000	4	40	2	20
	>30,000	3	30	5	50
Exercise habit	Yes	7	70	5	50
	No	3	30	5	50
Drinking habit	Never	6	60	8	80
	Sometimes	4	40	2	20
	≥3 days per week	0	0	0	0
	Daily	0	0	0	0
Smoking habit	Never	10	100	10	100
	Sometimes	0	0	0	0
	≥3 days per week	0	0	0	0
	Daily	0	0	0	0
Chronic illness	0	10	100	10	100
	1	0	0	0	0
	≥2	0	0	0	0
Dependency	Independent	10	100	10	100
	Dependent	0	0	0	0

M = mean, SD = standard deviation.

**Table 3:** Summary of outcome analysis for the research objectives.

Objective	Data Measurement	Result
To examine the effectiveness of the Connect Active Programme (CAP) on improving physical fitness.	6-Minute Walk Test (6MWT) (measured in minutes)	Statistically significant (*p* = 0.005 *)
To examine the effectiveness of the Connect Active Programme (CAP) on improving physical fitness.	Time Up and Go (TUG) (measured in seconds)	Statistically nonsignificant (*p* = 0.123)
To examine the effectiveness of the Connect Active Programme (CAP) on improving psychological well-being.	WHOQOL-BREF (Cantonese version)	Statistically significant (*p* = 0.015 *)
To examine the effectiveness of the Connect Active Programme (CAP) on improving psychological well-being.	Oxford Happiness Questionnaire	Statistically nonsignificant (*p* = 0.393)
To examine the effectiveness of the Connect Active Programme (CAP) on improving intergenerational relationships.	Intergenerational relationship quality scale (IRQS) of older participants	Statistically nonsignificant (*p* = 0.315)
To examine the effectiveness of the Connect Active Programme (CAP) on improving intergenerational relationships.	Intergenerational relationship quality scale (IRQS) of *younger participants*	Statistically significant (*p* < 0.001 *)
To examine the experiences and feedback from participants in using the apps and joining the CAP.	Mobile Application Rating Scale (MARS) (session-based only for the experimental group)	Rated below equilibrium

* *p*-value < 0.05 to be considered significant.

**Table 4:** CAP experimental group’s average Mobile Application Rating Scale scores.

Mobile Apps	N	Minimum	Maximum	Mean	Std. Deviation
Hong Kong Hiking Routes	10	1	4	2.10	0.876
Nike Run Club	10	1	4	1.80	1.033
WhatsApp	10	2	4	3.30	0.675
Google Map	10	1	4	2.70	1.059
Meitu	10	1	2	1.30	0.483
MTR/KMB *	10	1	3	2.10	0.738
Flower Companion	10	1	2	1.20	0.422
Valid N (listwise)	10				

* Note: Mass Transit Railway (MTR); Kowloon Motor Bus (KMB).

## Data Availability

The datasets used and/or analysed during this current study are available from the corresponding author upon reasonable request.

## Supplementary Materials

The following supporting information can be downloaded at: https://www.mdpi.com/article/10.3390/healthcare13162043/s1, Table S1: Implementation Fidelity Checklist (Week 1−6).

## Abbreviations

CAP	Connect Active Programme
PA	Physical Activity
