# Supplementary material for: Connect Active Programme (CAP): A Pilot RCT to Enhance Physical Activity and Intergenerational Relationships Through Dyadic Digital Walking Exercises
Source: Healthcare (Basel). 2025 Aug 19;13(16):2043. doi: 10.3390/healthcare13162043 (PMC12385363; doi:10.3390/healthcare13162043)
Supplement: Supplementary file 1 [file healthcare-13-02043-s001.zip › healthcare-3766181-supplementary.pdf]

healthcare

MDPI

Table S1. Implementation fidelity checklist (week 1–6).

Week 1: Walking in the Sports Ground		
**Delivery Content**	**Technology used**	**Assessment**
☐ Familiarize the older participants with the Nike Run Club App for exercise recording in the subsequent sessions.<br>☐ Teach the older participants to use Google Maps to search for routes leading to a destination. Use the sports ground as a starting point and introduce the surrounding community facilities to the older participants.<br>☐ Aid older participants in performing <u>3 physical assessments.</u><br>☐ Timed Up and Go Test<br>☐ 6-Minute Walk Test<br>☐ 30-minute 2km walking exercise tracked by the Nike Run Club App	☐ Nike Run Club App<br>☐ Google map<br>☐ KMB / LWB / MTR mobile app	**<u>Both Generations:</u>**<br>☐ Demographics (older participants)<br>☐ Demographics (younger participants)<br>☐ Intergenerational Relationship Quality Rating Scale (IRQS) (older participants)<br>☐ Intergenerational Relationship Quality Rating Scale (IRQS) (younger participants)<br>**<u>Older Participants Only:</u>**<br>☐ Pain self-efficacy questionnaire (Chinese version)<br>☐ Timed Up and Go Test<br>☐ 6-Minute Walk Test<br>☐ WHOQOL-BREF (Cantonese version)<br>☐ Oxford Happiness Questionnaire
**Week 2: Walking in the Park**		
**Delivery Content**	**Technology used**	**Assessment**
<u>Waterfront Park</u><br>Route: Waterfront Park chosen by participants<br>**Venue:**<br>____________________<br><u>During the walks</u><br>☐ Participants shall use the interactive app 'Flower Companion,' which is an app that identifies surrounding plants using a mobile phone camera.<br>☐ Young participants can introduce the concept of digital misinformation or health misinformation during walking.<br>☐ Brainstorming question example: when identifying the flower, can we rely on a single source? Then, guide the seniors to search 「viral」 in https://annielab.org/ to reveal a real case of misinformation.<br>Remarks:<br>____________________	Basic digital skills:<br>☐ Nike Run Club<br>☐ Flower Companion Mobile App<br>Cybersecurity:<br>☐ Fact-checking free resources: Annie Lab https://annielab.org/ ,<br>☐ HKBU Fact Check website<br>☐ https://factcheck.hkbu.edu.hk/home/	☐ Mobile Application Rating Scale (Flower Companion)<br>☐ Mobile Application Rating Scale (Nike Run App)
**Week 3: Walking in the Museum**		
**Delivery Content**	**Technology used**	**Assessment**
<u>Arts in Hong Kong</u><br>Route: Museum chosen by participants<br>Venue:<br>____________________	Basic digital skills:<br>☐ Nike Run Club<br>☐ Meitu Mobile App<br>☐ WhatsApp	☐ Mobile Application Rating Scale (Meitu)<br>☐ Mobile Application Rating Scale (WhatsApp)

Delivery Content	Technology used	Assessment
<u>During the walks</u><br>☐ Participants will use the mobile phone camera to take photos.<br>☐ Make a video or edit the photos of their visit with the Meitu App<br>☐ Send the photos or videos to friends or relatives.<br>☐ Younger participants can introduce the concept of privacy when using digital media, for example, photography ethics. When taking photos, do not encroach on people's space or be mindful of things around you, as they will be part of your shot's background; ask others before posting a group photo on social media.	Cybersecurity:<br>☐ Introduction of potential privacy infringement	
**Week 4: Walking in the Childhood Neighborhood**		
**Delivery Content**	**Technology used**	**Assessment**
Route: Designed by the older participants to visit places from their childhood<br>Venue: ____________________<br><u>During the walks</u><br>☐ The older participants shall use public transportation apps such as the KMB/LWB app or MTR app with the assistance of the younger participants to search for appropriate transportation and routes to travel to the "old places."<br>☐ The older participants can introduce their memories and stories of living in their childhood **neighborhood** to the younger participants while walking through these places.<br>☐ Explore the old town more via ChatGPT.<br>☐ Cybersecurity reminder example: Do not upload personal information when using the ChatGPT (Microsoft Bing) and reinforce fact-checking for older adults.	Basic digital skills:<br>☐ Google map<br>☐ KMB / LWB / MTR app<br>☐ Microsoft Bing free ChatGPT.<br>Cybersecurity:<br>☐ Introduce the risk of privacy intrusion when using the Internet.	☐ Mobile Application Rating Scale (Google map)<br>☐ Mobile Application Rating Scale (KMB / LWB / MTR app)<br>☐ Mobile Application Rating Scale (Microsoft Bing free ChatGPT)
**Week 5: Hiking in Country Parks**		
**Delivery Content**	**Technology used**	**Assessment**
Route: Chosen by the older participants<br>Venue: ____________________<br><u>During the hike</u><br>☐ Participants can use apps like "Hong Kong Hiking Routes" to track their trips.<br>☐ Younger participants: When seeing any QR code when walking, introduce what a QR code is and how to use it to the	Basic digital skills:<br>☐ Hong Kong Hiking Routes App<br>☐ Introduction of QR code<br>Cybersecurity:<br>☐ Introduction of Phishing link	☐ Mobile Application Rating Scale (Hong Kong Hiking Routes App)

elderly; if there is no QR code throughout the trip, brainstorm questions like 'Do you still remember we need to scan the LeaveHomeSafe QR code? Do you think it was convenient?' can be asked to initiate the discussion.<br>☐ A QR code is a link that can lead to phishing incidents. Beware of and do not click suspicious links. Share a video of ADCC during rest https://www.adcc.gov.hk/zh-cn/video-detail/video-1566958190368923649.html		
**Week 6: Walking in the Neighborhood**		
**Delivery Content**	**Technology used**	**Assessment**
Route: Neighborhood community chosen by participants<br>Venue: ____________________________<br>During the walk<br>☐ Younger participants can introduce the community resources for cybersecurity, e.g., the ADCC website.<br>☐ Take a photo of their neighborhood and then send it to relatives or friends via WhatsApp to introduce the place	☐ Reinforce technology and cybersecurity information.	**Both Generations:**<br>☐ Intergenerational Relationship Quality Rating Scale (IRQS) (older participants)<br>☐ Intergenerational Relationship Quality Rating Scale (IRQS) (younger participants)<br>**Older Participants Only:**<br>☐ Pain self-efficacy questionnaire (Chinese version)<br>☐ Timed Up and Go Test<br>☐ 6-Minute Walk Test<br>☐ WHOQOL-BREF (Cantonese version)<br>☐ Oxford Happiness Questionnaire
